# Molecular Analysis of the Processes of Surface Brown Spot (SBS) Formation in Pear Fruit (*Pyrus bretschneideri* Rehd. cv. Dangshansuli) by De Novo Transcriptome Assembly

**DOI:** 10.1371/journal.pone.0074217

**Published:** 2013-09-18

**Authors:** Pu Liu, Cheng Xue, Ting-ting Wu, Wei Heng, Bing Jia, Zhenfeng Ye, Li Liu, Liwu Zhu

**Affiliations:** Key Laboratory of Pomology, School of Horticulture, Anhui Agricultural University, Hefei, People’s Republic of China; University of Southern California, United States of America

## Abstract

Browning disorder, which usually occurs post-harvest in pears subjected to long-term storage, can cause browning of the pear flesh and/or core. In 2011, investigators in China found a novel type of brown spot (designated as surface brown spot, SBS) in pre-harvest ‘Dangshansuli’ pears (*Pyrus bretschneideri* Rehd.). SBS has a large impact on the exterior quality of the pears. Interestingly, the brown coloration was only found on the peel and not the flesh or the core. In this paper, *de novo* transcriptome analysis of the exocarp of pears with SBS using Illumina sequencing showed that SBS up-regulated the expression of genes related to oxidative phosphorylation, phenolic compound synthesis and polyphenoloxidase (PPO), and SBS was associated with inhibition of primary and secondary metabolism genes. Ca^2+^-sensor proteins might be involved in the signal transduction that occurs during the process of SBS formation, and this signaling is likely to be regulated by H_2_O_2_, abscisic acid (ABA) and gibberellic acid (GA_3_). Phytohormone and mineral element analyses confirmed that GA_3_, ABA, H_2_O_2_ and Ca^2+^ contribute to SBS formation. In addition to the seasonal characteristics, low levels of O_2_ and Ca^2+^ in the fruit are potential causes of the browning response due to exposure to oxidative stress, oxidative-reductive imbalance and the accumulation of reactive oxygen species (ROS), which affected the membrane integrity. Disruption of the membranes allows for PPO and phenolic compounds to come into contact, and the phenolic compounds are oxidized to form the browning pigments.

## Introduction

*Pyrus bretschneideri* Rehd. is widely distributed in northern China, and it is a popular variety due to its unique fragrance, desirable taste, sweetness and high digestibility [1]. *P. bretschneideri* Rehd. cv. Dangshansuli (also known as ‘Suli’) is the most important commercial Asiatic (Oriental) pear cultivar, and it accounts for 25% of the total annual pear yield in China. Recently, ‘Dangshansuli’ became the first pear cultivar with a fully sequenced genome [2].

Browning disorders (BDs) usually occur during the long-term storage of pears and apples. BDs can cause great economic losses, especially in controlled atmosphere (CA) storage [3]–[4]. This physiological disorder is characterized by browning of the flesh, cavities, core, etc. The symptoms are internal and cannot be observed visually without cutting the fruit in half [5]. Many studies, especially in the case of ‘Conference’ pears (*P. communis* L), have revealed that BD is usually induced by adverse storage conditions (low temperature, low O_2_, and high CO_2_) post-harvest, an imbalance between the oxidative and reductive processes and the accumulation of reactive oxygen species (ROS) [5]–[7]. Components of the antioxidant system, particularly L-ascorbic acid (L-AA), protect the fruit against browning through the neutralization of ROS to H_2_O [7]–[8]. Meanwhile, the pre-harvest conditions also affect the development of BD, including the growth temperature, rainfall, tree and soil characteristics, irrigation and geographical position [5]. Plant hormones, including abscisic acid (ABA), salicylic acid (SA), gibberellin (GA) and ethylene, are also involved in the BD processes [9]–[10].

**Figure 1:**
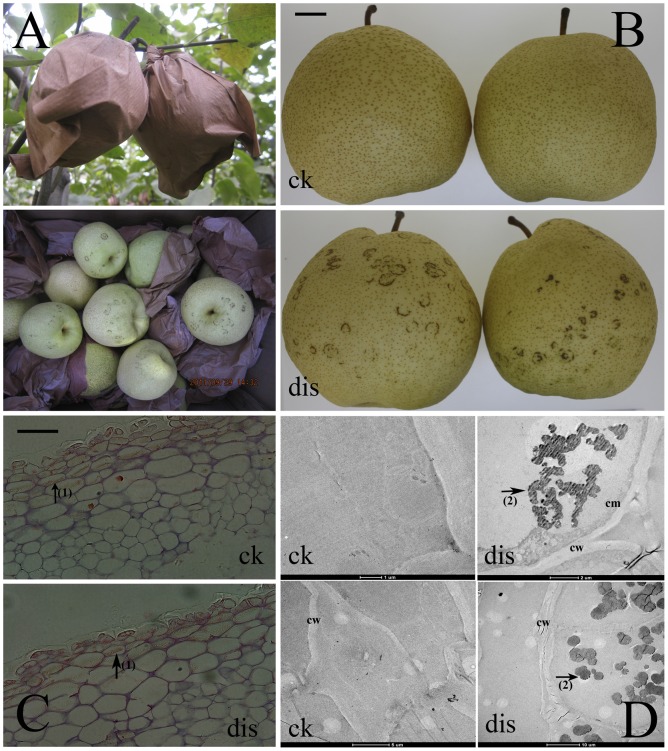
Surface brown spots disease (SBS, dis) in “Dangshansuli” pear fruit. (A) The SBS pears were collected from the orchards at which the fruit was bagged. (B) SBS Symptoms. The dark brown spot is confined to the peel of the fruit, and other parts of fruit, such as the flesh and core, are normal. (C) Paraffin sections observed by optical microscopy. (D) Transmission electron microscope (TEM). Arrow1: lignin; arrow 2: polyphenolic bodies. Bar: 2 cm.

Many papers have been published on the role of phenolic compounds and polyphenoloxidase (PPO) in the development of browning [11]. The occurrence of BD is due to the enzymatic oxidation of phenolic compounds by PPO to quinones and the formation of brown-colored polymers. Therefore, the PPO activity and phenolic compounds are leading factors in BD formation [5]. Phenolics, such as flavonoids, are mainly derived from the phenylpropanoid pathway. Fruit quality is based on a combination of attributes, properties and characteristics that result in the commodity value of the fruit as a human food. Consumers judge the quality of fresh fruits on the basis of appearance, firmness, flavor and nutritional quality. To improve the commercial value of pear fruit, pre-harvest bagging (Figure 1A) has been extensively used in both Asian and European pear varieties, such as ‘Dangshansuli’, ‘Yali’ (*P. bretschneideri* Rehd.), ‘Huangguan’ (*P. bretschneideri* Rehd.), ‘Conference’ pears (*Pyrus communis* L.) and ‘Concorde’ (*P. communis* L.) [12]–[13].

In 1998, a novel surface brown disorder (SBS) (also known as Jizhua disease) was identified in ‘Huangguan’ during post-harvest storage. This disorder is characterized by the appearance of irregular browning symptoms, and the spots have a semicircular type appearance. Initially, the affected areas of the skin are light brown in color, but as the disorder progresses, the skin becomes dark brown. Interestingly, this disorder only affects the peel of the fruit, and the other parts (flesh and core) are normal. The incidence SBS on ‘Huangguan’ pears was 10% in 2003 for Jin County of Hebei Province, and it was 15% in 2004 for all of Hebei Province. In 2010, all pear orchards Zhao County of Hebei Province reported cases of SBS pre-harvest. Recently, this new disease has arisen in ‘Dangshansuli’, affecting 26%, 54% and 30% of the pre-harvest pears in three counties of Shanxi Province (Pingyao, Qi and Wenshui). Compare to ‘Huangguan’, SBS in ‘Dangshansuli’ appeared as regular browning symptoms, and the spots are shaped with a ring (Figure 1B). In addition, SBS was also found in other cultivars of pear, such as ‘Xuehua’ (*P. bretschneideri* Rehd), ‘Xueqing’ (*P. pyrifolia* Nakai), ‘Suisho’ (*P. pyrifolia* Nakai) and ‘Lubaoshi’ (*P. pyrifolia* Nakai). SBS seriously impacts the exterior quality of pear. Limited research in China has mainly focused on the cultivar ‘Huangguan’. Bagging, Ca^2+^ content, temperature, sunshine and rainfall have been found to influence the occurrence of SBS [14]. Exogenous treatment with 1-MCP and CaCl_2_ can significantly reduce the incidence of SBS [15].

Transcriptomic, proteomic profiling and metabolic analyses have provided rapid methods of identifying and profiling differentially expressed gene and protein expression in fruit in response to stress [3], [16]–[17]. However, relatively little is known about the molecular mechanisms that regulate SBS processes in the pear, especially in the case of ‘Dangshansuli’. In the present study, genome-wide *de novo* transcriptome profiling was performed using a high-throughput sequencing platform (Illumina HiSeq™ 2000) to better understand the molecular mechanisms of SBS formation in the pre-harvest orchards.

## Materials and Methods

### Ethics Statement

China has established the National Research Organization called “China Agriculture Research System (CARS)” since 2008, which include 50 agriculture industries and the China Pear Research System is one of them. Prof. Liwu Zhu had been selected as a Scientist of CARS (work in China Pear Research System) since 2008 in charge of the field of Pear Cultivation under Adverse Circumstance and been responsible to studies of relevant pear cultivation problems occurred at any area in China.

The sampling site, a farmer pear orchard in Qi County, Shanxi Province, China was a demonstration base of China Pear Research System. In September 2011, a novel disease had occurred on ‘Dangshansuli’ pear fruit before harvested. Under invitation by Ms. Huangping Guo (Institutes of Pomology, Shanxi Academy of Agricultural Sciences, China), a director of Taigu Research Station, China Pear Research System and with the permission of the land owner, a team of five experts was sent by Prof Shaolin Zhang (Nanjing Agricultural University, Nanjing, China), the Chief-Scientist of China Pear Research System, to the venue for the practical investigation, it was named as Surface Brown Spots (SBS) by the expert team after the practical examination.

According to the study responsibility of Prof. Liwu Zhu’s Scientist position in China Pear Research System, ‘Dangshansuli’ pear fruit of SBS was harvested there and was carried to the Key Lab of Pomology, School of Horticulture, Anhui Agricultural University, Hefei, China for the further molecular analysis.

We confirm that the field studies did not involve endangered or protected species and there were no any vertebrate included in these studies.

### Fruit Material and RNA Extraction

‘Dangshansuli’ pear fruit with SBS was harvested a farmer pear orchard in Qi County, Shanxi Province, China and transported to Anhui Agricultural University, Hefei, China. The peel (exocarp) of pears exhibiting SBS was directly collected and ground into a powder in liquid nitrogen and stored at −80°C. Total RNA was extracted using TRIzol regent (Invitrogen, USA) according to the manufacturer’s instructions. Samples of 20 µg of total RNA were prepared for Illumina sequencing (*de novo* transcriptome assembly). Transcription was carried out essentially as described in previous studies [18]. Briefly, poly(A) mRNA was isolated with oligo (dT) beads and treated with fragmentation buffer to degrade the mRNA into short fragments. The RNA fragments were used as templates to synthesize the first-strand cDNA using random hexamer-primer, followed by treatment with RNase H and DNA polymerase I to synthetize second-strand cDNA. Purified short fragments were connected with sequencing adapters and selected for the PCR amplification. The cDNA library was sequenced using Illumina HiSeq™ 2000.

### Transcriptome Data Analysis

To remove the reads that contained the adaptor, unknown or low-quality bases, a filter was applied to generate clean reads. *De novo* assembly of the short reads to contigs and unigenes was carried out using the assembly program, Trinity [19]. Unigenes from the assembly of each sample were further processed for sequence splicing and redundancy removal using sequence clustering software to acquire non-redundant unigenes of the longest possible length. Functional annotation was performed by comparing our obtained unigenes with protein databases such as NR, Swiss-Prot, KEGG and COG blastx (E-value<1.0e^−5^). When a unigene was found that was unaligned with the above databases, ESTScan software [20] was be used to predict its sequence direction and coding regions. The expression level of each unigene was estimated using the RPKM method (Reads per kb per Million reads). Differentially expressed genes (DEGs) in two samples were analyzed as described [21]. Then, the DEGs were submitted to a Gene Ontology (GO) functional analysis and Kyoto Encyclopaedia of Genes and Genomes (KEGG) pathway analysis. The GO categorization results are expressed as three independent hierarchies for biological process, cellular component and molecular function. The biological interpretation of the DEGs was further completed by assigning them to metabolic pathways using KEGG. For the identification of pathways significantly affected by SBS, we focused on the metabolic pathways with at least three affiliated genes [22]–[23].

### Real-time Quantitative RT-PCR (qRT-PCR) Verification

Total RNA was reverse transcribed into first-strand cDNA using the M-MLV First Strand kit (Invitrogen, USA) according to the manufacturer’s instructions. Twenty-two genes were chosen for confirmation by qRT-PCR with SYBR Premix Ex Taq™ (Takara, Japan). Primers for the chosen genes were designed with the Primer Express software (Applied Biosystems, USA) and are presented in Table S1. qRT-PCR for gene expression analysis was performed on a StepOne Real-time PCR System (Applied Biosystems, USA) using glyceraldehyde-3-phosphate dehydrogenase (GAPDH) gene as an endogenous control according to [24]. Briefly, the primers for the target gene and GAPDH were diluted in the SYBER Mix (Applied Biosystems) and 20 µL of the reaction mix were added to each well. The reactions were performed with an initial incubation at 50°C for 2 min and at 95°C for 1 min followed by 40 cycles of 95°C for 15 s and 60°C for 1 min. The levels of gene expression were analyzed with StepOne Software v2.0. Zero template controls were included for each primer pair. Each PCR reaction was carried out in triplicate, and the data are presented as the means ± SD.

### Plant Hormones, H_2_O_2_ and Mineral Element Contents Analysis

A 10-g sample (fresh weight; FW) of the exocarp was ground into a powder in liquid nitrogen. Quantification of endogenous ABA, indole-3-acetic acid (IAA), zeatin-riboside (ZR) and gibberellic acid (GA_3_) were analyzed using the enzyme-linked immunosorbent assay (ELISA) method, which was performed as described in [25]. The concentration of H_2_O_2_ was assayed using H_2_O_2_ assay kits (Nanjing Jiancheng Bioengineering Institute, China) according to the manufacturer’s instruction. The mineral elements (Ca, Fe and K) in the exocarp of the fruit were analyzed as described in [26].

### Microstructure Analysis

Small pieces of peel were cut from the diseased zone in pears with SBS. The tissues were fixed overnight in formalin-aceto-alcohol (FAA) and then dehydrated through ethanol to 70% for long-term storage. The fixed tissues were embedded in wax, and sections (9 µm) were stained with safranin O. High-resolution images of the pear peels were captured using a microscope (Olympus BX51).

Transmission electron microscopy (TEM) was performed according to the following standard procedure. Briefly, small tissues (1 cubic millimeter) were fixed with 5% glutaraldehyde for 24 h in the dark and washed with 0.1 M PBS (pH 7.2) three times (15 min each). The samples was postfixed in reduced osmium tetroxide (2% OsO_4_) for 8 h and washed three times in PBS. The fixed tissues were dehydrated in a standard ethanol series (20 min each) and embedded in Spurr’s low-viscosity resin for 12 h at 45°C. Semithin and ultrathin sections (40–60 nm) were cut with a Leica EM UC7 (Leica Microsystems, Vienna, Austria), mounted on regular hexagonal copper grids, stained with lead citrate (10 min), washed three times with de-carbon dioxided deuterium-depleted water (ddw), stained with uranyl acetate for 30 min and washed with ddw three times. The thin sections were examined using an FEI Tecnai F20 transmission electron microscope operating at 200 KV.

### Total Phenolic Analysis

A 3-g powder sample (FW) was extracted with 15 mL methanol, mixed with a vortex for 1 min, followed by centrifugation and filtration through a 0.45-µm cellulose membrane filter. The filtered supernatants were evaporated and re-dissolved in methanol for analysis. The total concentration of phenols in the extract was determined according to the Folin-Ciocalteu method [27].

## Results

### Characteristics of Fruit with SBS Compared with Controls

As shown in Figure 1, SBS was found at the orchards where the bagging of the fruit occurs. SBS was only found on the peel of the pear, and not on the flesh or core. Tissue observation showed that the presence of lignin (Figure 1C arrow 1) and the number of cell layers differed between SBS fruit and control (CK). Using TEM, a large number of bodies containing homogenous substances with a similar density and appearance to those of polyphenolic bodies were visualized (Figure 1D arrow 2). These bodies might be responsible for the brown color of the peel.

### Illumina Sequencing and Assembly of the Reads

To isolate DEGs in the SBS pear fruit and compare the samples with those collected from CK, cDNA libraries were constructed using RNA for Illumina sequencing. In total, 23 and 25 million clean reads of 90 bp were obtained from the SBS and CK fruit, respectively, after filtering the reads. Raw data are deposited on NCBI SRA, accession numbers: control SRR891358 and SBS SRR891594. The *de novo* assembly of the remaining clean raw reads was carried out using the Trinity assembly program [19]. We obtained 106,965 contigs with a mean length of 310 bp for the CK fruit and 107,710 contigs with an average length of 300 bp for the SBS fruit (Table 1). Then, the reads were mapped back to the contigs. With paired-end reads, it was able to detect contigs from the same transcript as well as the distances between these contigs. Finally, Trinity connected the contigs and obtained sequences that cannot be extended on either end. The contigs were assembled into 53,668 CK-unigenes with a mean length of 614 bp (N50∶1045 bp) and 49,632 SBS-unigenes with a mean length of 622 bp (N50∶1039 bp). Using sequence clustering software, 46,472 non-redundant unigenes (all-unigene) with a mean size of 805 bp were obtained from the clean CK and SBS reads combined. The length distribution and gap distribution of the unigenes from the three combinations (CK, SBS and CK&SBS) are shown in Figure S1. The CK-unigenes and SBS-unigenes had a similar distribution in length. In total, the all-unigenes were better than the other two types of unigenes with regard to quality, and this set had higher identity scores when using BLASTx to search against the nonredundant (NR) NCBI nucleotide database. In total, 33,583, 30,658, 20,233, 18,500, 12,009 and 13,915 of the unigenes were functionally annotated with the NR, NT, SwissProt, KEGG, COG and GO databases, respectively (Table S2 and S3).

**Table 1:** Summary of the transcriptome reads for the normal control (CK) fruit and fruit with surface brown spots disease (SBS).

	CK	SBS	SBS & CK
Total reads collected	25,578,784	28,000,000	
Total clean reads	23,461,256	25,446,658	
GC percentage	47.36%	48.93%	
Number of contigs	106,965	107,710	
Mean length of contigs (bp)	310	300	
Number of unigenes	53,668	49,632	
Mean length of unigenes (bp)	614	622	
Number of singletons	51,033	46,904	
Number of all-unigenes			46,472
Mean length of all-unigenes			805

### Unigene Analysis of Differential Expression between SBS and CK Fruit

**Figure 2:**
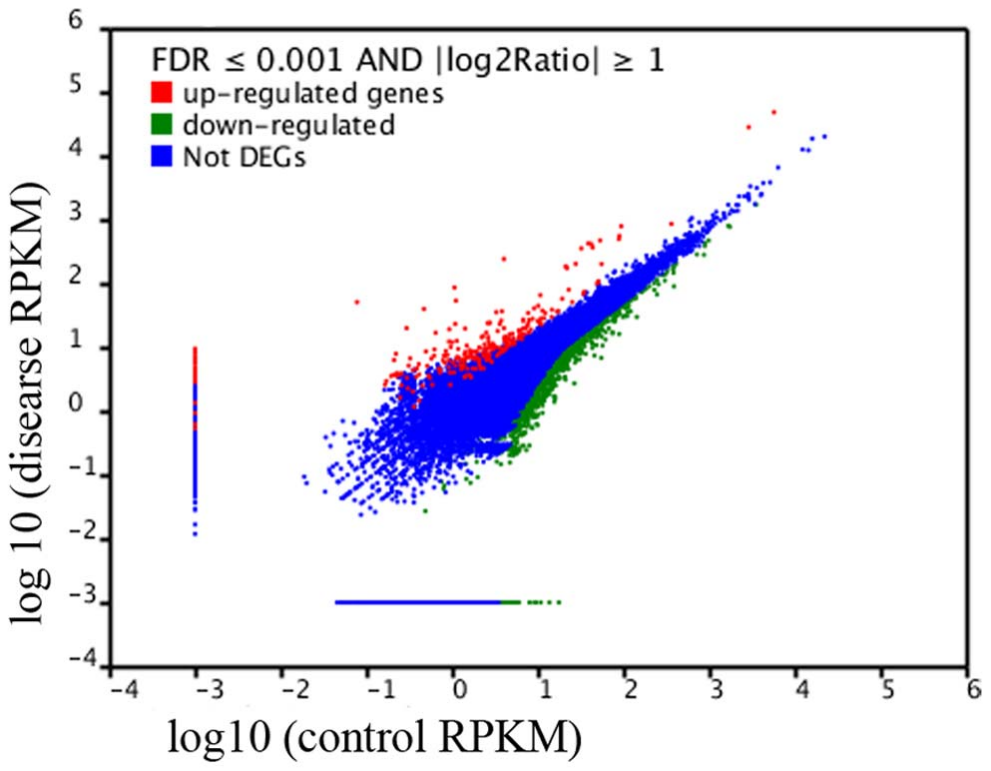
Comparison of unigene expression between the normal control (CK) and surface brown spots disease (SBS, dis) fruit. The abundance of each gene was normalized as Reads per kb per Million reads (RPKM). The differentially expressed genes are shown in red and green, while blue indicates genes that were not differentially expressed (not DEGs) between CK and SBS.

**Figure 3:**
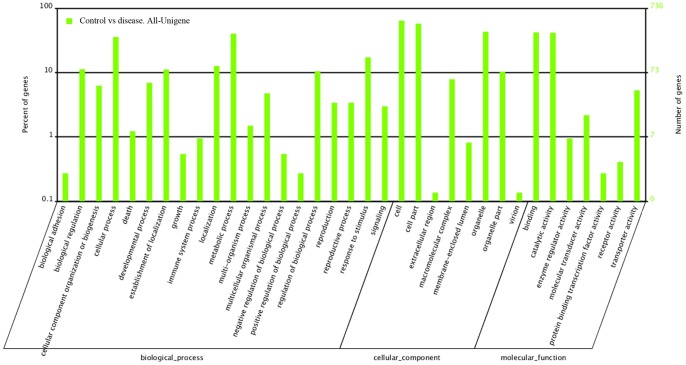
Functional categorization of the genes with significant transcriptional changes between the normal control (CK) and surface brown spots disease (SBS, dis) fruit. The genes were categorized based on Gene Ontology (GO) annotation, and the proportion of each category is displayed based on the following: Biological process; Molecular function; Cellular component.

The relative expression level was investigated using the RPKM method [21], which can eliminate the influence of differences in the gene length and sequencing level for the calculation of gene expression. Comparative analysis of the signature frequencies between CK and SBS revealed that the expression ratio (SBS/CK) varied greatly from 0.0331 to 662. A general picture of the gene expression was plotted for the SBS fruit compared with the CK fruit (Figure 2). Only 1.96% of the signatures were species specific, in that they were found only in one library and were absent from the other. Analysis of differential expression in SBS fruit compared to CK fruit revealed 2431 down-regulated genes (ratio >2; FDR≤0.001) and 258 up-regulated genes (ratio <0.5; FDR≤0.001). GO categories were assigned to the 2689 DEGs using the Blast2GO program (http://www.blast2go.org) to evaluate the potential functions of the DEGs between the CK and SBS fruit. The DEGs were finally categorized into 20 groups on the basis of their biological processes, as shown in Figure 3. Cellular process (263), metabolic process (296), localization (93), response to stimulus (127), biological regulation (83), establishment of localization (82) and regulation of biological process (77) were the major categories. Categories based on the cellular component revealed that the SBS-responsive genes were mainly related to cell (472), cell part (423) and organelle (318). With regard to molecular function, the DEGs were classified as binding (309), catalytic activity (306), enzyme regulator activity (7), molecular transducer activity (16) and transporter activity (39). Of these, plastid, intracellular organelle and membrane were the major sub-cellular organelles involved in the response to SBS.

The SBS-responsive genes were further assessed using KEGG pathway analysis. A total of 15 different metabolic pathways were found with more than 3 affiliated genes, of which some were consistent with biological processes that were already identified by GO analysis. The most represented pathways are listed in Table 2. Most of these were related to BD formation based on previous knowledge, including oxidative phosphorylation (25), metabolic pathways (230), flavonoid biosynthesis (17), stilbenoid, diarylheptanoid and gingerol biosynthesis (15), biosynthesis of secondary metabolites (99), flavone and flavonol biosynthesis (6), limonene and pinene degradation (11), linoleic acid metabolism (5), monoterpenoid biosynthesis (4) and α-linolenic acid metabolism (10).

**Table 2:** List of the important KEGG pathways with more than 3 differentially expressed genes.

KEGG Pathway	Genes	Gene ID
Photosynthesis	11	CL20193, CL20623, CL23584, CL2580, CL26681, CL2718, CL28160, CL955, Unigene13509, Unigene15889, Unigene1923
Oxidative phosphorylation	25	CL1072, CL1184, CL1324, CL20080, CL20623, CL23584, CL2488.Contig1l, CL29847, CL3545, CL3677, CL489, CL52, CL5226, CL5229, CL5961, CL759, CL8193, CL970, Unigene10550, Unigene10553, Unigene15505, Unigene3303l, Unigene5796, Unigene7506, Unigene9854
Flavonoid biosynthesis	17	CL21109, CL21498, CL26833 l, CL27252, CL28550, CL3827, CL8442l, CL8682, Unigene10998, Unigene11889, Unigene12756, Unigene13313, Unigene14636, Unigene15904, Unigene2652, Unigene7469, Unigene9848
Stilbenoid, diarylheptanoid andgingerol biosynthesis	15	CL15335, CL21109, CL21498, CL26833, CL5149, CL7772., Unigene10959, Unigene13468, Unigene14636, Unigene15904, Unigene2652, Unigene4655, Unigene4995, Unigene7469, Unigene8041
Flavone and flavonol biosynthesis	6	Unigene12756, Unigene14636, Unigene15904, Unigene2652, Unigene4845, Unigene9848
Limonene and pinene degradation	11	CL15335, CL5149, CL7772, Unigene10959, Unigene13468, Unigene14636, Unigene15904, Unigene2652, Unigene4655, Unigene4995, Unigene8041
Ether lipid metabolism	49	CL1032, CL1032, CL12521, CL13277, CL1508, CL15842, CL16149, CL1680, CL18686, CL18871, CL19411, CL20202, CL20295, CL21111, CL23863, CL25214, CL25809, CL26901, CL27690, CL27777, CL28086, CL28596, CL28688, CL29005, CL29121, CL29276, et al
Linoleic acid metabolism	5	CL20955, CL4220, Unigene2757, Unigene8508, Unigene9358
Monoterpenoid biosynthesis	4	CL15695, Unigene2012, Unigene2217, Unigene8791
Endocytosis	56	CL12521, CL13277, CL144.Contig3, CL14564, CL1508.Contig2, CL15842, CL16149, CL1680, CL18686, CL18871, CL19411, CL20202, CL20295, CL21111, CL23807, CL23863, CL25214, CL25809, CL26901, CL27690, CL27777, CL28086, CL28596, CL28688, CL29005, et al
α-Linolenic acid metabolism	10	CL10316, CL1116, CL20955, CL4220, Unigene1390, Unigene2596, Unigene2757, Unigene8508, Unigene913, Unigene9358

KEGG: Kyoto Encyclopedia of Genes and Genomes. Pathway enrichment of differentially expressed genes was analysis at P-value ≤0.05.

### Differential Expression of SBS Formation Genes between CK and SBS Fruit

BD is a common disease that occurs during CA storage of pears, and it is characterized by core breakdown, CO_2_-injury and brown core and heart. Both pre-harvest and post-harvest elements can lead to browning. To identify browning-related DEGs, a total of 2689 DEGs were assessed by BLAST searches against the NCBI NR, Swissprot and NT protein datasets. A number of new genes that are potentially related to browning were identified in this study. Function category analyses revealed that, in addition to the above pathway, 5 ABA-related DEGs (ABA hydroxylase, ABA responsive element binding factor), 3 GA_3_-related DEGs (gibberellin dioxygenase, gibberellin oxidase), 14 ethylene-related DEGs (ethylene responsive transcription factors) and 9 L-AA-related DEGs (L-ascorbate oxidase, ascorbate transporter, ascorbate oxidoreductase) were found between CK and SBS. In addition, 4 other antioxidant-related genes were down-regulated in SBS.

The second group of significant browning-responsive genes includes 20 calcium-related genes, including calcineurin B-like (CBL) proteins and CBL-interacting proteins kinases (CIPK), as well as boron (CL14381), which is consistent with previous reports that treatment of fruit with calcium and boron can reduce browning in ‘Conference’ and ‘Huangguan’ [28]. In addition, the PPO gene (unigene14299), an oxidase that catalyzes the oxidation of phenolic compounds to o-quinones, showed 3.8-fold up-regulation in SBS compared with CK fruit according to the Illumina sequencing data. Consistent results were found by qRT-PCR, which indicated that the gene expression of PPO in SBS was 3.7-times higher than in the CK fruit.

### Verification of the Brown Disorder-related Genes

**Figure 4:**
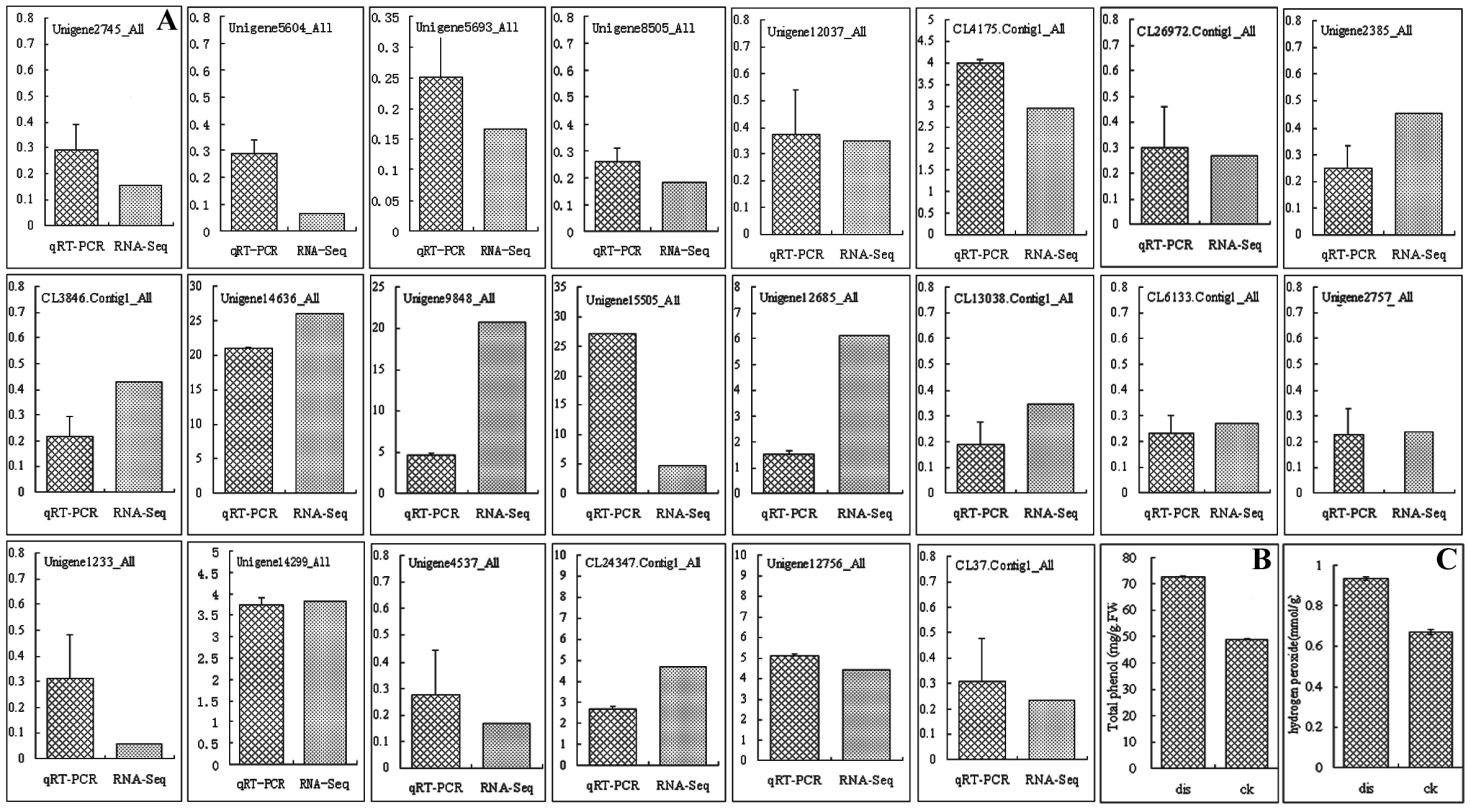
A: Real-time quantitative RT-PCR confirmation of the differentially expressed genes between normal control (CK) and surface brown spots disease (SBS, dis) fruit. B: The total phenolic content in CK and SBS fruit. C: H_2_O_2_ content in CK and SBS fruit. The total phenolic was measured using the Folin-Ciocalteu method. Columns and bars represent the means and standard error (n = 3), respectively.

To confirm that the DEGs identified by deep sequencing were indeed differentially expressed, a total of 22 genes were chosen for confirmation in a biologically independent experiment using qRT-PCR, including POD, calcium, ethylene and the ABA-related genes, which were detected in the transcriptome and bioinformatic analyses. The relative transcript abundance patterns for the CK and SBS fruit were compared using the transcriptome data. The results of qRT-PCR revealed the same expression patterns as the Illumina sequencing despite some quantitative differences in the expression levels (Figure 4A).

### Measurement of Hormones, H_2_O_2_, Total Polyphenols and Mineral Element Content

**Figure 5:**
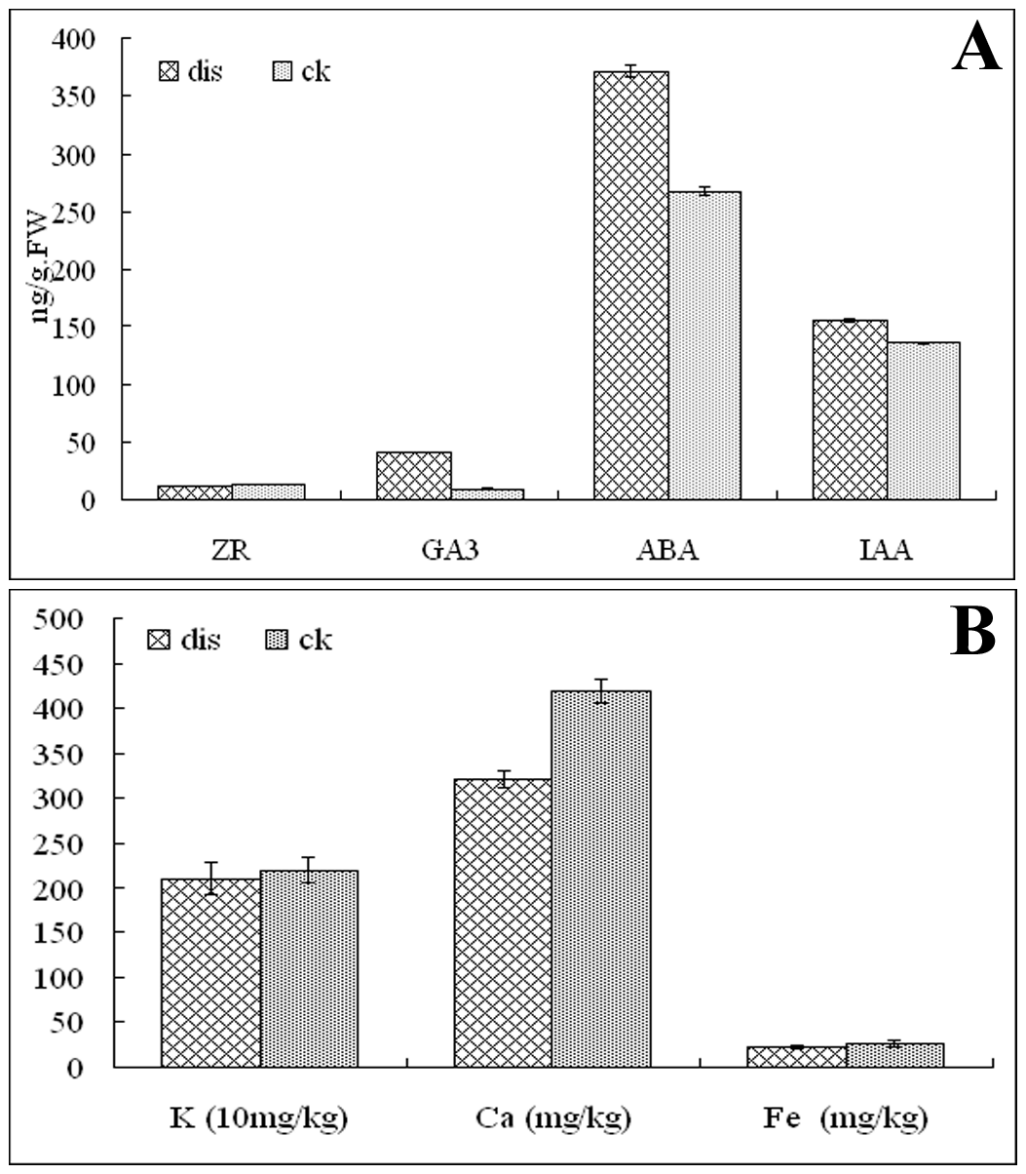
Phytohormone and mineral content differences in the normal control (CK) and surface brown spots disease (SBS) fruit. A: Phytohormone contents in the CK and SBS fruit; B: mineral element contents in the CK and SBS fruit. Phytohormones were measured using the ELISA method, and mineral elements were measured using atomic absorption spectrometry. Three independent repetitions for each test and 5 fruits from each sample were measured; the average values and standard deviations are displayed.

The ZR, ABA, GA_3_, IAA, H_2_O_2_, K, Fe and Ca content were measured in CK and SBS fruits with three repetitions. The measurements of the hormones ZR, ABA, GA_3_ and IAA revealed that the contents of ABA and GA_3_ were higher in SBS than in CK (Figure 5), and no significant differences were found for ZR and IAA between CK and SBS. Meanwhile, SBS fruit had an increased content of total polyphenol. These data are consistent with the results of the Illumina sequencing. Mineral element analysis of K, Fe and Ca revealed that the content of Ca was the most significantly different between CK and SBS, which is also in accord with the transcriptomic data. In fruit, H_2_O_2_ plays an important role in the hypersensitive response. In this study, we found that browning fruit had increased levels of H_2_O_2_ compared with the sound control.

## Discussion

Comparative study is an important and effective strategy for the critical analysis of genotypes of plants with different levels of stress tolerance. BD is commonly observed in fruits and vegetables. Recently, a novel browning disorder was detected in pear fruit and designated as SBS. Although non-coding RNAs (such as miRNA) can control the transcription and translation of proteins, mRNA as protein-coding RNA directly carries information from about a protein sequence to the ribosomes, the protein synthesis factories in the cell, so we directly analysis the genes for mRNA by ployA enrichment method. In this study, we used the Illumina sequencing method to monitor the global transcription changes in the pears with SBS compared with CK pears. We identified 2689 DEGs at a 0.05% significance level that were induced or repressed by more than two fold in the SBS fruit. A large number of newly discovered and interesting unigenes encoding transcription and post-transcription factors were included in these DEGs, indicating that these genes may be key regulators that control browning by activating or repressing numerous genes. Additionally, a number of putative homologs of genes for SBS were also found.

**Figure 6:**
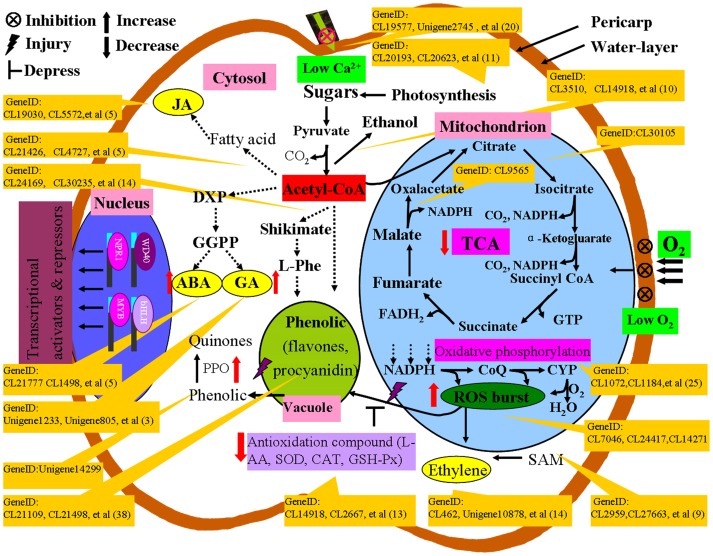
Overview of the major metabolic pathways involved in brown spots disease (SBS). The genes are designated according to their gene ID number, and the detailed gene information can be viewed in additional file Table S3. JA, jasmonic acid, ABA; abscisic acid; GA, gibberellic acid; SAM, S-adenosyl methionine; PPO, polyphenol oxidase.

The first noticeable pathway is the phenolic biosynthesis pathway, which exists in the vacuole. In total, 38 differential genes are involved in phenolic biosynthesis (flavone, flavonoid, and flavonol), including CL8682 (encoding flavonol synthase), Unigene8485 (encoding isoflavone reductase) and CL3296 (encoding flavanone 4-reductase) (Figure 6). In addition, 14 different phenylpropanoid biosynthesis and 8 different tyrosine metabolism genes were also found in our study. The role of phenolic compounds in plant physiology has been known for many years, including procyanidin and chlorogenic acid [29]. These compounds are mainly derived from phenylalanine via the general phenylpropanoid pathway [30]–[31]. Two dihydroflavonol reductase (DFR) genes were up-regulated (CL28550 and CL3296); DFR genes that catalyze the formation of the precursor (leucocyanidin) for anthocyanidins, catechins and procyanidins. Meanwhile, Cytochrome P450 plays an important role in flavone biosynthesis and has been well characterized. Over 18 different cytochrome P450 genes were detected in our Illumina sequencing data. The transcription levels of Unigene14636 (isoflavone 2′-hydroxylase, CYP82C4), Unigene12756 (flavonoid 3-hydroxylase, CYP75A2) and Unigene9848 (flavonoid 3′-monooxygenase, CYP82A1) in SBS that were detected by Illumina sequencing were 26, 20 and 4 times higher than in the control, respectively, which is consistent with our qRT-PCR results (Figure 4). Based on these data, it can be concluded that the phenolic biosynthesis genes are up-regulated during pear fruit browning.

Previous studies have reported that the browning phenomenon is usually attributed to the enzymatic reaction of the phenolic compounds, causing the formation of quinines [32]–[34], such as chlorogenic acid, which is oxidized to chlorogenic acid quinine by PPO. Meanwhile, peroxidase (POD) is also able oxidize a wide range of phenolic compounds to produce ortho-diquinones [35]–[36]. Highly reactive quinones then react spontaneously and nonspecifically to polymerize proteins and other cellular components into the amorphous form of brown pigments (Figure 1D). In this study, one PPO gene (Unigene14299) and two POD genes (CL24417 and CL13038) were detected by Illumina sequencing. PPO was 3.8-fold up-regulated in the SBS fruit compared with the control according to the Illumina sequencing data. The qRT-PCR data were consistent, demonstrating that PPO in SBS was 3.7-times higher than in CK, indicating that the oxidation of phenolic compounds may be regulated by PPO.

It is interesting that ABA and GA_3_ catabolism were affected in the SBS fruit. Three ABA catabolism genes (ABA 8′-hydroxylase, CL26972, CL3846 and CL1498) and two GA_3_ catabolism genes (gibberellin 2-oxidase, Unigene1233 and Unigene805) were down-regulated. All of these findings fit well with our observation that the ABA and GA content in the SBS fruit was higher than that in the control. The enhancement of ABA and GA_3_ levels in SBS may be the result of a reduction in the degradation of ABA and GA_3_. Therefore, our data support the hypothesis that ABA and GA_3_ are regulators of the browning formation processes in SBS. In pineapple, endogenous ABA and GA_3_ influence the internal browning of fruit [10].

Normally, PPO and phenolic compounds are located in different cell compartments (cytoplasm/plastids and vacuole, respectively) [5], [37]. PPO and phenolic compounds come into contact when this compartmentation is broken, resulting in the oxidation of phenolic compounds. Previous studies have shown that disruptions of oxidation and reduction may induce BD, leading to ROS bursts, which would affect the membrane integrity [5]–[7]. The second group of significant SBS-responsive pathways includes photosynthesis, the citrate cycle (TCA cycle), oxidative phosphorylation and fatty acid metabolism (Table 2 and Figure 6). Changes in these metabolic processes will disrupt the energy metabolism of the fruit, providing a molecular context for the formation of ROS in SBS.

The third group of metabolic pathways involved in SBS occurs downstream of the antioxidation pathway. In total, 13 genes involved in antioxidant biosynthesis were down-regulated, including L-AA, superoxide dismutase (SOD), catalase (CAT) and glutathione peroxidase (GSH-Px) biosynthesis. A number of papers have reported on the role of the antioxidant system in the development of BD. It has been shown that L-AA protects against browning and that browning does not occur unless the L-AA concentration falls below a certain threshold value [5]. Usually, SOD, CAT, POD and GSH-Px have a synergic effect with L-AA. These enzymes neutralize ROS by converting them to H_2_O. Phenolic compounds seem to protect the fruit by scavenging ROS (Figure 4), but the corresponding brown-colored oxidation products are the actual cause of the browning symptoms [7].

Several families of transcription factors, including MYB, bHLH (basic helix-loop-helix) and WD40 proteins, interact and establish transcriptional networks to control the general phenylpropanoid and phenolic compound biosynthetic genes [30]. It is noteworthy that several families of transcriptional factors, including the MYB gene, showed significant transcriptional changes in the SBS fruit as revealed by the Illumina sequencing data. Furthermore, qRT-PCR analysis confirmed the results, suggesting that these transcriptional factors play a role in control the formation of browning pigments. However, to the best of the author’s knowledge, it is still unknown whether these transcriptional factors regulate phenolic compound oxidation. This study identified an important gene that possibly regulates the accumulation of browning pigments in the peel of pear fruits.

Bagging, high humidity and rainfall disrupt the absorption of calcium by the pear fruit due to depressed transpiration. Previous data showed that post-harvest calcium application limited the BD occurrence in cold storage [38]. As a second messenger of the host signaling pathway, calcium is an important component of the communication system that operates between the cytoplasm and the cell wall. When the level of Ca^2+^ is too low, the wall is weakened and may even break. In this study, a number of Ca^2+^-sensor protein families, including calmodulin (CaM), the superfamily of calcium depended protein kinases (CDPKs), calcineurin B-like (CBL) proteins and CBL-interacting proteins kinases (CIPK), have been identified as being down-regulated in SBS, and analysis of the Ca^2+^ content confirmed the results. In *Arabidopsis thaliana*, the cbl9 mutant demonstrated that AtCBL9 modulates ABA sensitivity and biosynthesis [39]. The biological functions of CIPKs appear to be linked to ion channel activities, stress and/or stress responsive processes (ABA, drought, cold, salts). For example, CBL4 together with the interacting protein kinase CIPK6 modulates the activity of the K^+^ channel AKT2 [40]. Meanwhile, Ca^2+^ and ROS are second messengers that exhibit cross-talk. Enhancing the intracellular Ca^2+^ level results in the activation of ROS and antioxidant enzymes. On the other hand, an increase in intracellular Ca^2+^ concentration can also be triggered by ROS [41].

Combine with data on seasonal characteristics (Figure S2), we predict that bagging results in a particular set of changes in intrinsic pear attributes (cuticle wax, Ca^2+^, phenolics content) that may be leading factors in SBS formation. On the other hand, rainfall, high humidity and temperature are inducers of SBS occurrence, although the Pearson correlation coefficient does not reach a conspicuous level (P>0.05, α = 0.05). One possibility is that rainfall and high humidity produce a water film (or bubble) around the fruit, which reduces the gas transport ability [42], and lowers the intercellular O_2_ and oxidative stress of fruits, causing an oxidative-reductive imbalance and an ROS burst. Meanwhile, bagging, rainfall and high humidity depressed fruit Ca^2+^ absorption and utilization. ROS and Ca^2+^ cross-communicate and work collaboratively in the development of fruit SBS.

## Conclusion

Our study provides a global picture of the gene expression changes in SBS compared with a sound control. The transcriptomic data revealed a large number of genes that were previously not known to be involved in the process of browning formation. Functional categorization of the DEGs revealed the involvement of a number of important pathways, including oxidative phosphorylation, photosynthesis, the TCA cycle, phenolic compound synthesis, calcium signaling, hormone cross communication and some transcription factors, in regulating the browning process.

## Supporting Information

Figure S1 **The length distribution of unigene.**
(TIF)

Figure S2 **Seasonal characteristics of the orchard.**
(TIF)

Table S1 Primers used for real-time quantitative RT-PCR for the verification of Illumina data. Optimal oligonucleotide sequences for real-time RT-PCR were predicted by primer express program to prevent faint PCR products as primer dimmer and false amplicon.
(XLS)

Table S2 The functionally annotated with the Gene Ontology.
(XLS)

Table S3 All differential expression between SBS and CK fruit.
(XLS)
