# Supplementary figures and images for: Molecular Analysis of the Processes of Surface Brown Spot (SBS) Formation in Pear Fruit (Pyrus bretschneideri Rehd. cv. Dangshansuli) by De Novo Transcriptome Assembly
Source: PLoS One. 2013 Sep 18;8(9):e74217. doi: 10.1371/journal.pone.0074217 (PMC3776823; doi:10.1371/journal.pone.0074217)

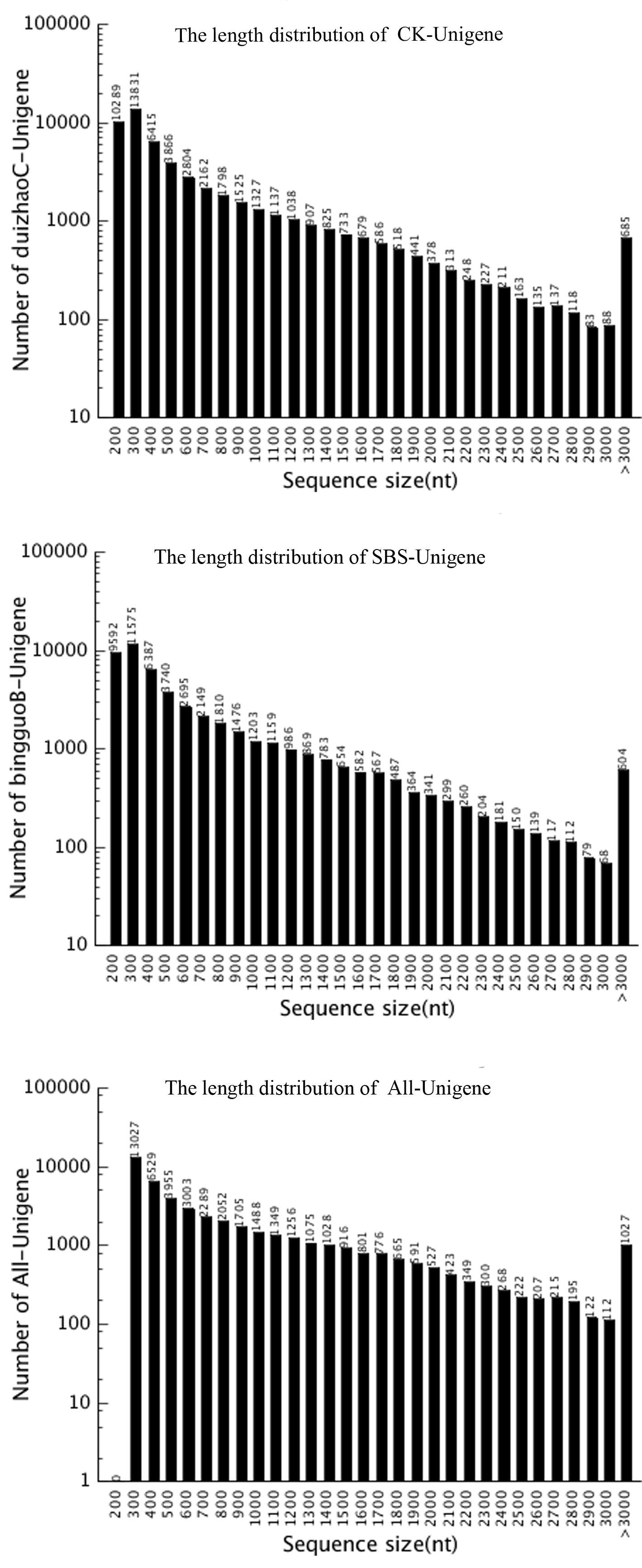

Supplement: Figure S1 — The length distribution of unigene. (TIF) [file pone.0074217.s001.tif]

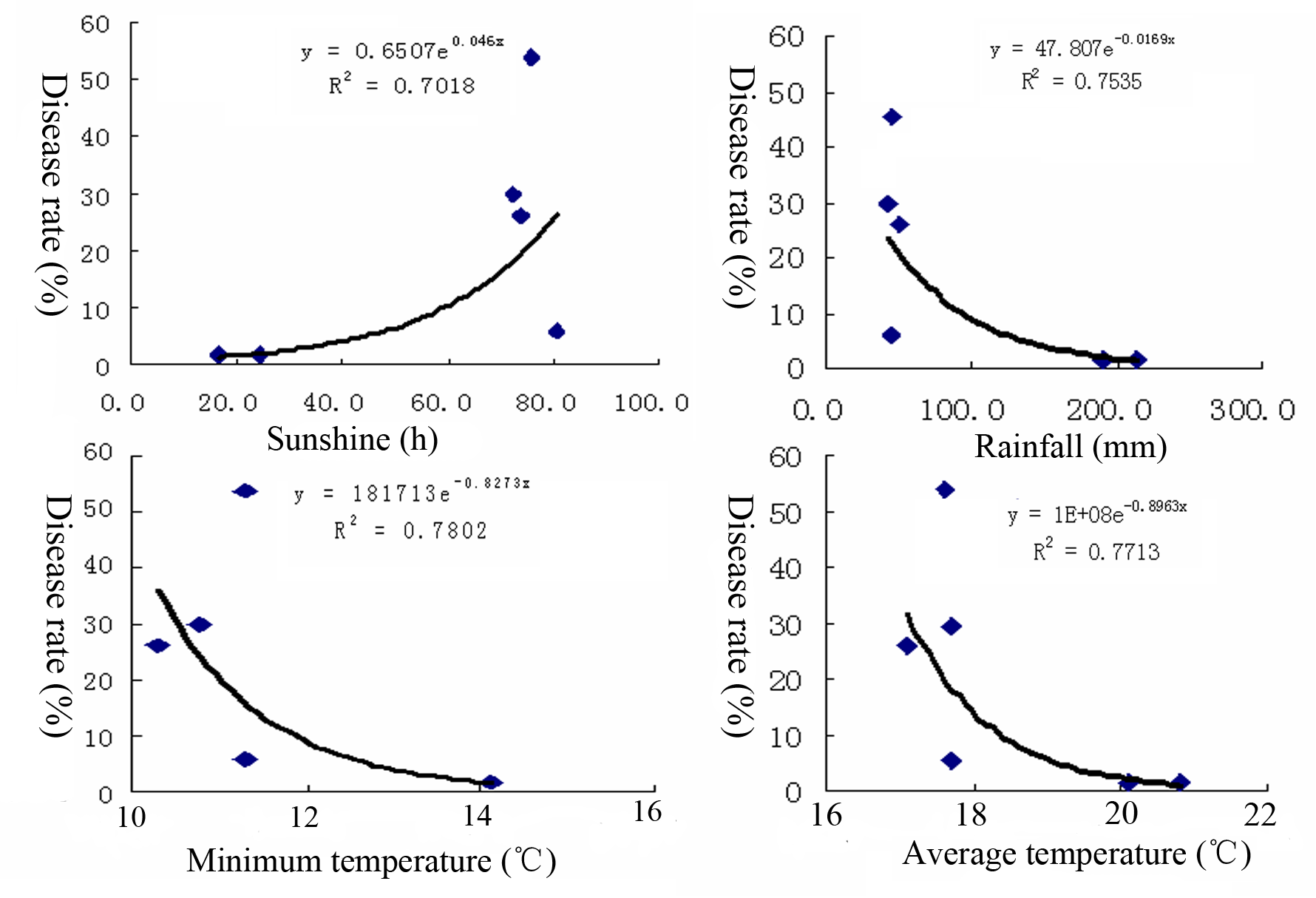

Supplement: Figure S2 — Seasonal characteristics of the orchard. (TIF) [file pone.0074217.s002.tif]
